# Hybrid Ablation of Nonparoxysmal Atrial Fibrillation: The Convergent and Totally Thoracoscopic Approaches

**DOI:** 10.31083/j.rcm2310338

**Published:** 2022-10-11

**Authors:** HelenMari Merritt-Genore, Armin Kiankhooy

**Affiliations:** ^1^Methodist Physicians Clinic, Omaha, NE 68022, USA; ^2^Department of Cardiothoracic Surgery, Saint Helena Hospital, Saint Helena, CA 94574, USA

**Keywords:** hybrid ablation, arrhythmia surgery, CONVERGE, totally thoracoscopic maze, Cox-Maze surgery, left atrial appendage, pulmonary vein isolation, posterior wall isolation

## Abstract

Nonparoxysmal atrial fibrillation continues to challenge electrophysiologist and surgeons alike. Stand-alone endocardial catheter ablation has resulted in less than satisfying results, and while the on-pump Cox-Maze surgery has excellent results, the invasiveness has limited its adoption amongst both referring providers and surgeons. The CONVERGE IDE trial has shed new light on this once dim problem. EPs and Surgeons are now working together in a Hybrid Team Ablation Approach to provide a combined ablation strategy that has improved patient outcomes and rekindled the collaboration necessary to better patient outcomes. We herein summarize the current Hybrid Team Ablation Approaches (CONVERGE and Totally Thoracoscopic) with nonparoxysmal atrial fibrillation.

## 1. Background

Atrial fibrillation (AF) is a global health concern with over 43 million people affected worldwide as of 2016, and substantial expected growth over the coming decades [1]. The burden of AF begins within months of diagnosis and may be best broken down into two categories: patient and economic factors. Patients with AF have increased risk of stroke, heart failure and notably up to a 46% greater risk of death [2, 3]. In addition to decreased quality of life, a rising concern is the association of decreased cognitive function and vascular dementia [4, 5, 6]. Atrial fibrillation is also costly. Annual patient costs are over $8700 *per year* as compared to patients without AF, and within the United States AF is estimated to cost the healthcare system over $26 billion annually [7, 8].

A comprehensive approach to the management of AF begins with addressing the basics and common co-morbidities of the disease (i.e., obesity, hypertension, excessive alcohol, sleep apnea and thyroid dysfunction), and a combined collaborative heart team approach. While the primary goal of medical therapy is to manage the symptoms of AF, medications are also utilized to decrease the incidence of thrombosis, and to restore normal sinus rhythm (NSR). Often, these medications fail or are poorly tolerated (either anticoagulation *or*antiarrhythmics), or patients prefer alternative strategies. The importance of pulmonary vein isolation has been clearly established through a mountain of research and is a mainstay of catheter ablation (CA) therapies but has limited effectiveness as a stand-alone therapy for non-paroxysmal atrial fibrillation (NPAF) which includes long-standing or persistent AF. For example, for paroxysmal AF a catheter ablation may provide up to an 80% freedom from AF (with multiple CAs), whereas even with multiple CAs addressing the pulmonary veins alone, the success rate for maintenance of NSR was closer to 45% for non-paroxysmal AF [9].

In addition to the pulmonary veins, the posterior wall of the left atrium is derived from the same embryologic tissues and is a common region for atrial fibrillation triggers [10]. Although recognized increasingly as a key component of NPAF management, endocardial CA in this region is often difficult due to the large area of substrate, and adjacent structures [10, 11].

Contemporary surgical management of stand-alone AF will be discussed in this article, with a key focus on two techniques which address the common triggers for AF: The Hybrid (epicardial and endocardial ablation) Convergent (HC) and the Hybrid Totally Thoracoscopic (HTT) or video-assisted thoracoscopic surgery (VATS) “Maze”. While each procedure addresses similar lesion sets, the incisions and endocardial versus epicardial lesions differ (Table 1).

**Table 1.** **Overview of HC and HTT surgical ablation approaches**.

	HC Surgical Ablation	HTT Maze
Endocardial Portion	Pulmonary Vein Isolation, mitral and coronary sinus lesions	Mitral and Tricuspid Isthmus Lesions
Surgical Portion	Subxiphoid Window: Posterior left atrial wall	Bilateral VATS: Bilateral pulmonary veins, coumadin ridge, Ligament of Marshall, partial mitral isthmus, coronary sinus, and right atrial lesions, appendage management
	Left VATS*: Appendage ligation, Ligament of Marshall	
Appendage Management?	If left VATS is performed*	Yes, routine
Complications*	Pericardial effusion, excessive bleeding, stroke, death	Phrenic nerve injury, vascular Injury, stroke, conversion to open incision, death
Strengths	Technical ease for most surgeons	More complete epicardial lesion set
Considerations	Appendage management performed in 80% of contemporary cases*	Technically more difficult surgically. Morbid obesity or poor pulmonary reserve may exclude candidates

*Most of the existing data for the HC approach has not included appendage management and thus a paucity of data exists for the increased risk or benefit when added to the HC procedure.

## 2. Hybrid Convergent Procedure

**Fig. 1.**
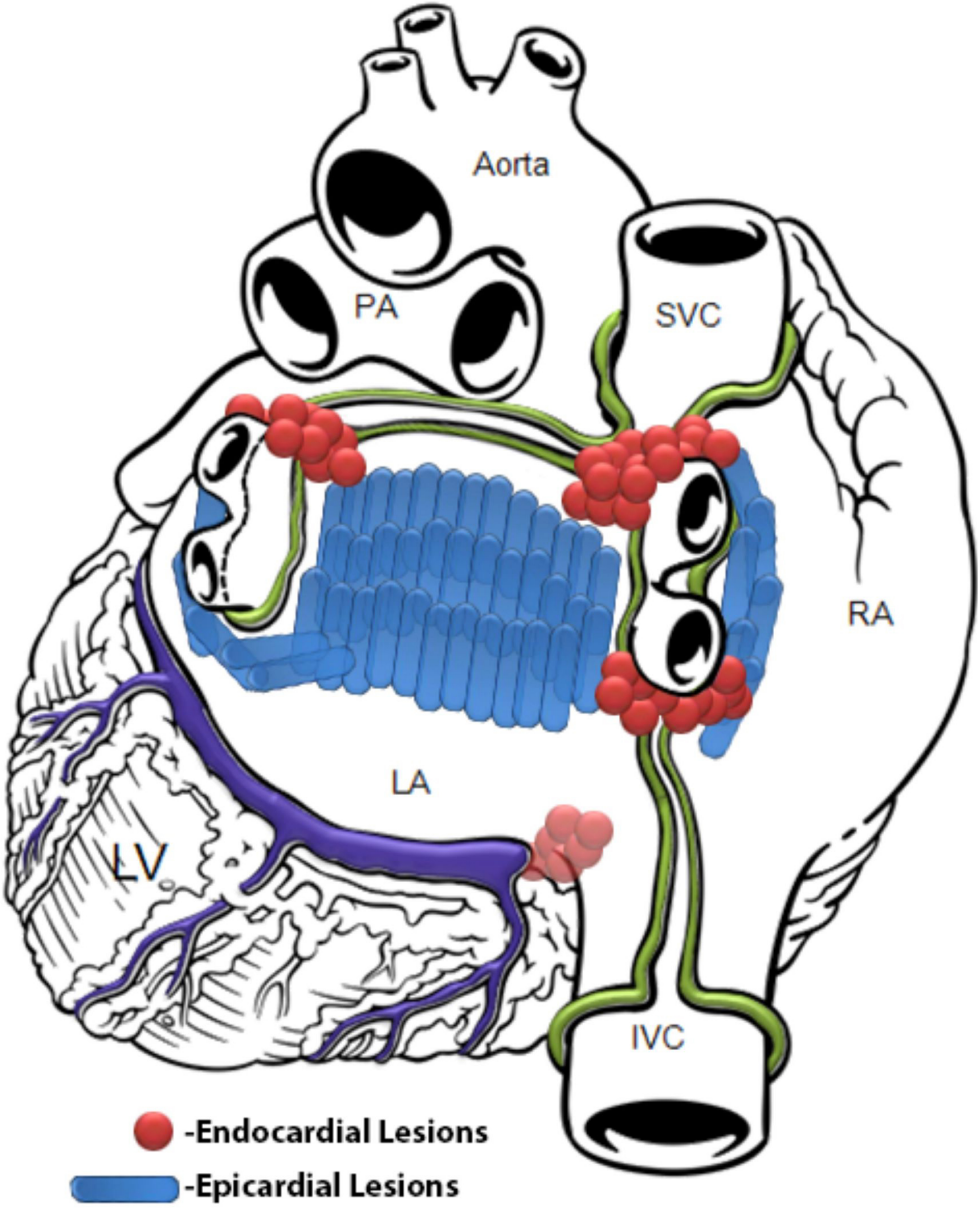
**Current standard HC lesion set (permission received from *AtriCure™*)**.

The Hybrid (epicardial and endocardial ablation) Convergent (HC) procedure addresses both pulmonary veins and the posterior left atrial wall (and often the caval-tricuspid isthmus as well). HC was first developed in the early 2000’s through collaboration between electrophysiologists and cardiothoracic surgeons, to expand non-sternotomy options for patients with atrial fibrillation resistant to initial strategies (medications and catheter ablation) [12]. Subsequently, there have been continued adaptations of the HC procedure due to new technology and modification of lesion sets. In some practices, hybrid surgical ablation may be considered a first line approach. The HC procedure has evolved from an extra-cardiac Cox-Maze III [13] lesion set to a series of parallel ablations lines across the posterior left atrial wall (Fig. 1). The hybrid approach likewise has changed from an initial laparoscopic transdiaphragmatic approach to a more familiar sub-xiphoid pericardial access.

### 2.1 The Convergent 1st Stage Epicardial Ablation Surgical Considerations

**Fig. 2.**
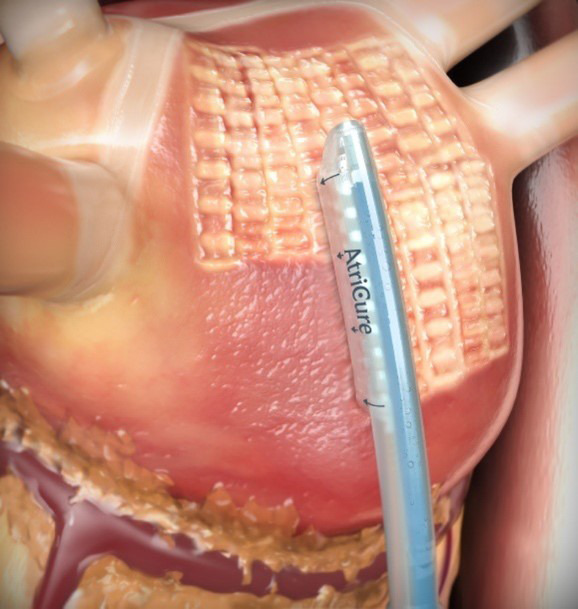
**Epicardial Convergent Procedure Ablation (permission received from *AtriCure™*)**.

There are several manuscripts which outline the step-by-step procedural steps of the 1st stage epicardial Convergent procedure in detail [14, 15]. In this portion of the manuscript, we will focus more on discussion points and less on the step-by-step operative approach. A TEE is performed to rule out the presence of left atrial or appendage thrombus. A temperature probe is placed into the esophagus as position behind the left atrium. A subxiphoid incision is made and the pericardium is entered in the standard fashion, as close to the diaphragmatic surface as possible. The presence of significant adhesions is excluded. Under direct endoscopic visualization the Convergent cannula is placed into the pericardial space, and landmarks are identified: the coronary sinus and the right and left inferior pulmonary veins. The unipolar radiofrequency ablation catheter is then advanced via the cannula and onto the epicardial surface of the posterior left atrium and ablation is performed, monitoring esophageal temperature and feedback from the device regarding the quality of the ablation (Fig. 2). Upon completion of all accessible territories of the posterior left atrial wall, a drain is placed, and the device and cannula are removed. The incision is closed in the standard fashion.

### 2.2 2nd Stage Endocardial Catheter Ablation Technique for Convergent Procedure

**Fig. 3.**
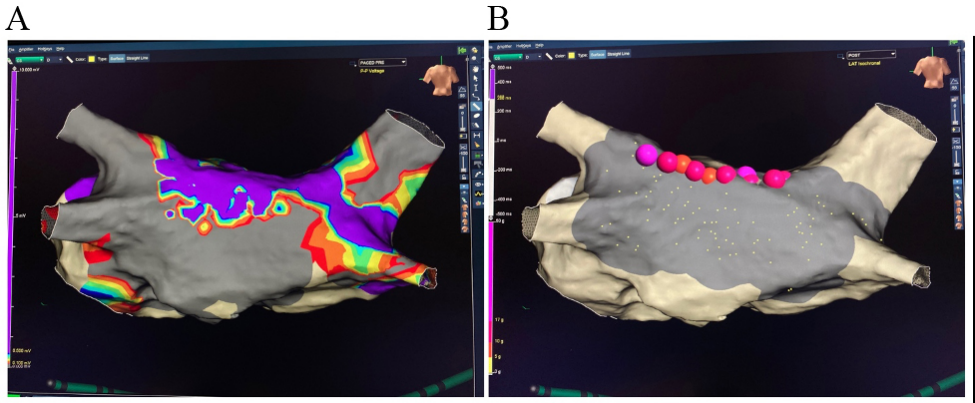
**Completed hybrid convergent procedure: posterior wall and bilateral pulmonary vein isolation**. (A) Post-1st Stage Epicardial ablation – prior endocardial Left-PVI. (B) Post-2nd Stage Endocardial ablation (Cryoballoon Right-PVI and Roof Radiofrequency ablation).

The second stage of the procedure is endocardial mapping and ablation. Even if patients have been previously ablated, there is potential benefit in confirming there is no gap in prior ablations and ensuring transmurality of the surgical lesion set (Fig. 3). While many centers allow time for reduction in inflammation which may obscure true signals of effective ablations, some centers practice a comprehensive hybrid approach with both procedures taking place on the same day and often in the same hybrid suite. There has not been a study to date comparing these two approaches with the Convergent approach.

### 2.3 Left Atrial Appendage Management with the Convergent Procedure 

**Fig. 4.**
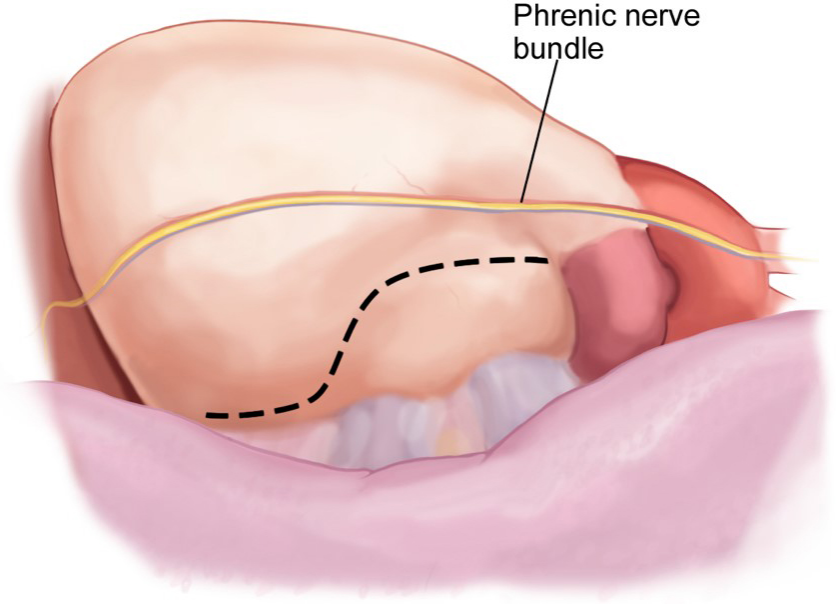
**Left atrial appendage access via a Left VATS pericardiotomy**.

If a concomitant appendage ligation is to be performed, the left lung is then deflated, and the TEE probe is reinserted. Again, the details of this portion have been previously well defined [14, 15] and will be only briefly reviewed for relevant pitfalls and controversial points. The phrenic nerve should be clearly identified, and the pericardium opened most commonly posterior to its course (Fig. 4). Caution should be used as the confluence of the PA and the pericardium is approached to avoid injury to this major vessel. Some surgeons will at this time ablate the Ligament of Marshall which lies here in the transverse sinus and may be easily obliterated with the use of vessel-sealing or ultrasonic energy devices. Once the base of the appendage is sized, the clip is advanced through the most inferior and posterior port. After the clip has been applied to the base of the appendage, an inspection in several views with TEE confirms a <1 cm remaining rim, and that there are no wall motion abnormalities or ST changes indicative of circumflex compromise. It should be noted that some programs prefer to address the appendage first to avoid reinsertion of the TEE probe.

### 2.4 Convergent Hybrid Procedure Data Review

The landmark trial for hybrid convergent ablation was the 2020 CONVERGE trial [16]. In this study, 153 patients were randomized 2:1 for hybrid approach versus standard catheter ablation (CA). This study was unique as it included patients with LA size up to 6 cm, and there was no limit on the duration of AF as compared with other studies which may have excluded longstanding persistent AF. In this study, 42% of patients had longstanding persistent AF. Eligible patients were those who were intolerant to at least one class I/III AAD, with symptomatic persistent AF.

The incidence of major complications (excessive bleeding, stroke, pericardial effusion) was low in both groups, but noted to be higher (5%) in the HC group. Patients should be monitored for pericardial effusion postoperatively. There were no deaths in either group in this study. Heart Rhythm Society (HRS) rhythm success is defined as freedom from atrial arrhythmia lasting >30 seconds [17]. HRS success at one year favored hybrid convergent (67% HC versus 50% CA, *p* = 0.036). Critics of these outcomes will quote the PRECEPT IDE trial [18], which demonstrated similar HRS success rates for CA alone (61.7% with 5.7% repeat ablation within blanking period); however, it should be noted that the duration of AF was limited in study with a mean duration of 15.9 months as compared to 4.4 years for the CONVERGE trial [16, 18].

While the BELIEF trial and the STAR AF II study showed marginal improvement in reduction of AF for CA endocardial posterior wall (and moderately increased rates of adverse events such as esophageal or nerve injury), the CONVERGE trial demonstrated a clear benefit for the hybrid approach with epicardial ablation of the posterior atrial wall. A 7-day Holter monitor was performed at 18 months and demonstrated a >90% reduction in AF burden which also favored hybrid convergent (74% versus 55%, *p *< 0.05) [16, 17, 19]. In addition, the Hybrid Convergent approach reduces the potential for esophageal injury as energy is applied away from the heart and towards the pericardium as compared to an endocardial approach.

A recent meta-analysis (2020) including 340 hybrid convergent patients demonstrated improved results at 1 year compared to catheter ablation but noted that results were not superior to the Cox-Maze IV procedure for HRS success in the prevention of recurrence of atrial fibrillation or overall avoidance of any complication. The complication rate for hybrid ablation in this study was 10% [20]. A second study confirmed these findings in a larger meta-analysis of 8 manuscripts including over 700 patients. Again, adverse events were noted to be higher in the hybrid convergent groups: 45 adverse events as compared to 17 events in the CA; 5 deaths as compared to zero [21]. The major critique of the bulk of research available on the hybrid convergent procedure is that the subxiphoid incision is now largely the primary approach, whereas the primary incision was transdiaphragmatic in these studies. The subxiphoid incision is a common, comfortable approach for cardiac surgeon, and obviates the need for laparoscopy, a skill with which many cardiac surgeons are less facile. The subxiphoid approach also avoids a transdiaphragmatic incision, which reduces any chance of herniation of abdominal contents into the pericardial space over time [21].

### 2.5 Convergent Procedure Summary

In summary, the HC ablation approach has the potential to improve long-term outcomes and decrease the burden of AF in patients with NPAF. The CONVERGE trial demonstrates safety and efficacy with a 5% risk of complications, even in patients with a long history of AF. The ideal patient will have poor tolerance to at least one anti-arrhythmic drug, and control of modifiable risk factors such as tobacco abuse and OSA. Prior cardiac surgery, severely decreased ejection fraction, and advanced age may be seen as a relative contraindication for the HC approach. These factors may vary based on surgeon experience. Poor pulmonary function, morbid obesity or prior lung procedures may preclude the addition of thoracoscopic appendage ligation.

## 3. Hybrid Totally Thoracoscopic Ablation Approach

In addition to the popularized Convergent hybrid ablation approach for non-paroxysmal atrial fibrillation (NPAF), surgeons and electrophysiologist have also developed the Hybrid Totally Thoracoscopic (HTT) or video-assisted thoracoscopic surgery (VATS) “Maze” approach for NPAF.

**Fig. 5.**
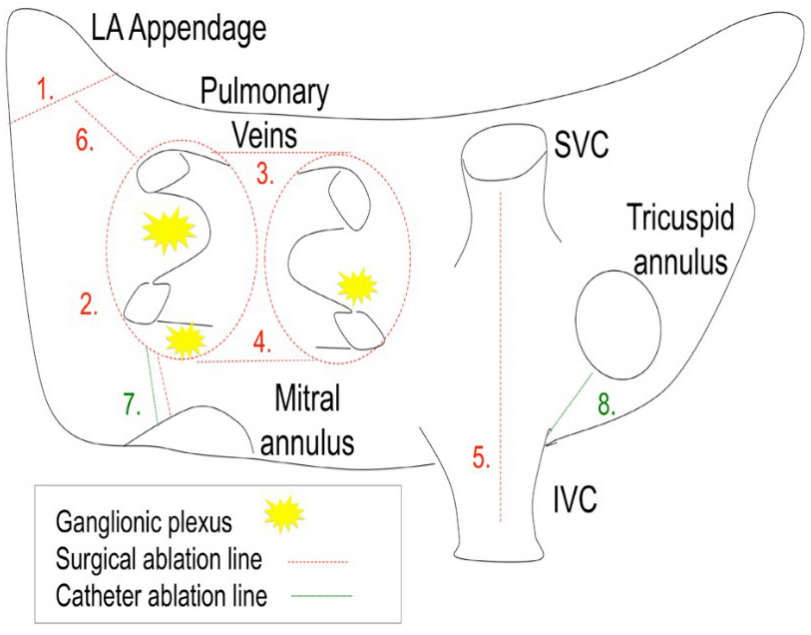
**Hybrid Totally Thoracoscopic (HTT) complete lesion set (permission granted from Eur J Cardiothoracic Surg) [22]**.

The HTT Maze is similar to the Convergent approach in that both epicardial and endocardial staged ablations are created to approach the Cox-Maze III/IV lesion set. However, the HTT Maze often includes additional lesions; including but not limited to division of the ligament of Marshall/vein of Marshall, epicardial bilateral pulmonary vein isolation(s), coumadin ridge ablation (left superior pulmonary vein to the left atrial appendage), partial mitral isthmus or coronary sinus ablations, and epicardial right atrial lesions (intercaval SVC-IVC lesion, Right atrial “T-Lesion” to the right atrial appendage) (Fig. 5) [22]. Therefore, in total the HTT Maze approach is intended to not only isolate the posterior wall (with standard interconnecting roof and floor lesions between bilateral pulmonary vein isolations) but also to target additional structures that may improve the overall patient response to a hybrid approach. The main limitation to greater adoption of the HTT Maze however has been the technical complexity of this surgical approach and the associated increase in patient risk. We herein, summarize the current published data about HTT Maze effectiveness and safety. Of note, the AtriCure sponsored DEEP IDE trial (Dual Epicardial Endocardial Persistent Atrial Fibrillation (AF) Study is ongoing and the results of this study are not available at the time of writing of this review.

### 3.1 The Totally Thoracoscopic 1st Stage Epicardial Ablation Surgical Technique

**Fig. 6.**
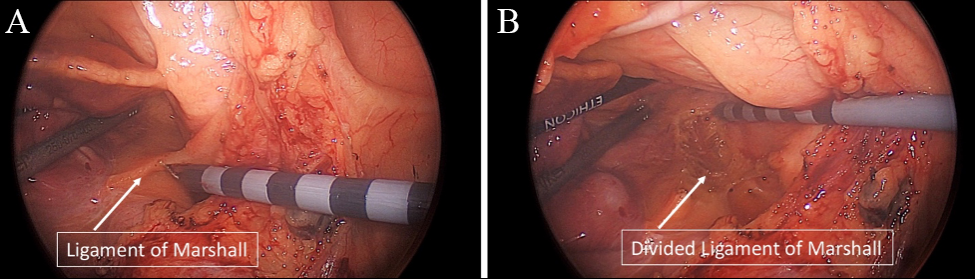
**Totally thoracoscopic mobilization of the anterior pericardial reflection and division of the ligament of Marshall**. (A) Ligament of Marshall (LOM) and Anterior pericardial reflection before mobilization and (B) after mobilization/ligation.

**Fig. 7.**
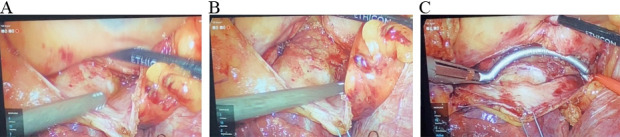
**Intra-atrial groove mobilization from the right chest during Totally Thoracoscopic ablation**. (A) Intra-atrial “groove” before mobilization, (B) after mobilization and (C) isolation with bipolar RF clamp.

The technical aspects of the HTT Maze are less familiar to most than the Convergent hybrid approach so we have highlighted the critical steps in the following section. The HTT Maze is currently most often performed under general anesthesia in the operating room (OR) via port-access. Surgeons typically begin with left lung isolation and access the epicardium via a left pericardiotomy posterior to the left phrenic nerve bundle, similar to the LAA approach during the Convergent procedure. At this point, direct visualization of the ligament of Marshall allows for direct division via electrocautery without complication. In rare cases, a vein of Marshall is encountered, but can be readily divided with electrocautery without adverse events. The use of bipolar cautery (i.e., Ligasure or Harmonic) may also be used to divide the ligament of Marshall and provides a safe and effective method for division of this tissue without the concern for collateral thermal injury. Next, the anterior pericardial reflection between the dome of left atrium and the left pulmonary artery is developed to enhance the ability to encircle the left pulmonary veins (Fig. 6). Once completed, a lighted-tip dissector (AtriCure) is used to encircle the left pulmonary veins and then guide the safe passage of a bipolar bi-directional radiofrequency clamp (AtriCure Synergy Clamp) to encircle the left pulmonary veins on the antrum of the left atrium away from the carina of the left pulmonary veins. Prior to ablation, the pulmonary veins are tested for the presence of entrance block. If epicardial isolation of the pulmonary veins is not present than successive ablations using the Synergy clamp are performed until successful isolation is confirmed with epicardial testing. After left pulmonary vein isolation, partial interconnecting lesions from the left pulmonary vein isolation across the roof and floor are performed using radiofrequency devices (i.e., AtriCure MLP or Coolrail). The interconnecting floor lesion between the left inferior pulmonary vein and right inferior pulmonary vein can often be completed, as visualization of the right inferior pulmonary vein is accessible in most patients. The interconnecting roof lesion between the left superior pulmonary vein and the right superior pulmonary vein, however, is often incomplete due to the fat pad between the dome of the left atrium and the superior pulmonary vein. This fat pad is most often developed from the right chest and can be readily completed at that time in order to complete the interconnecting roof line. After completion of the interconnecting lesions, the coumadin ridge lesion is created with radiofrequency (i.e., MLP) from the base of the left atrial appendage to the left superior pulmonary vein. Finally, some surgeons have also incorporated either a lateral mitral isthmus (coronary sinus to the left inferior pulmonary vein) or anterior mitral isthmus lesion (left superior pulmonary vein to the mitral annulus) to complete the left sided approach. Attention is then brought to left atrial appendage, which is then sized, and ligated with an AtriClip device (Pro-Clip 2 or V-Clip) in most instances. Placement and successful ligation of the left atrial appendage is confirmed with transesophageal echocardiography. This completes the left sided approach and can be accomplished in experienced hands in approximately 30–45 minutes. The remainder of the ablations are then performed from the right chest. Again, using right lung isolation and 3 or 4 port access, the right epicardium is visualized via a right pericardiotomy anterior to the phrenic bundle. The oblique and transverse sinuses are then developed to allow for complete access across the epicardium to the left side of the heart in order to visualize the prior partial interconnecting lesions. The right pulmonary veins are then epicardially tested in a similar fashion and ablated with the Synergy clamp to achieve electrical isolation. Importantly, the interatrial groove (Waterson’s groove or Sonegards groove) is developed to again aid with placement of the Synergy clamp on the antrum of the left atrium away from the carina of the pulmonary veins (Fig. 7). Completion of the interconnecting roof and floor lesions are then accomplished with radiofrequency energy to complete the posterior wall isolation. Finally, intercaval superior vena cava and inferior vena cava lesions can be completed as well right atrial free wall “T-lesions” and lesions to the right atrial appendage. In experienced hands, the right chest lesions require approximately 30–45 minutes also. The right sided pericardium is then reapproximated to prevent right atrial herniation post-operatively.

### 3.2 2nd Stage Endocardial Catheter Ablation Technique for Totally Thoracoscopic Procedure

**Fig. 8.**
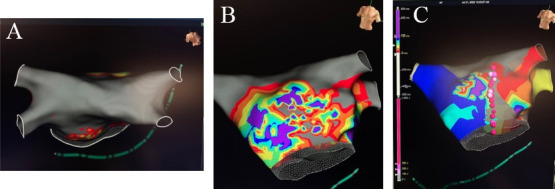
**Completion of the Hybrid TT Maze**. (A) Endocardial map after 1st stage Totally Thoracoscopic ablation with complete isolation of bilateral pulmonary veins and posterior wall with (B) partial epicardial anterior mitral line and (C) endocardial anterior mitral line completion.

The completion of the HTT Maze approach is performed via an endocardial catheter ablation in a similar fashion to the Convergent hybrid approach. Endocardial mapping is performed to identify gap areas in the epicardial lesions that require additional endocardial ablation and/or to identify the mitral and tricuspid isthmus for additional ablation (Fig. 8). With the addition of these endocardial lesions, the complete hybrid approach lesion set closely approaches the fundamental Cox-Maze III/IV. The most common reported endocardial energy applied is radiofrequency, however cryoballoon has also been utilized [23].

The HTT approach, like the Convergent procedure, can be completed either during the same hospitalization (in most instances a single anesthesia) or separated by 4–6 weeks at another hospitalization. That being said, whether the HTT Maze is completed via a two staged epicardial and endocardial approaches separated by 4–6 weeks or during a single hospitalization does not appear to lead to a significant difference in either rhythm control effectiveness or risk. As Dr. LeMair and colleagues [24] eloquently articulate in their article entitled, “The 7 Pillars to a successful hybrid program”, often hospital logistics determine the appropriateness of a single stage versus a two-stage approach. Moreover, groups that have internally compared their single hospitalization vs separated hospitalization HTT Maze approaches have found no statistically significant differences in rhythm outcomes [22, 25, 26]. However, it is important to note that when stages are separated via the standard 4–6 weeks it may allow for a clearer delineation of post-surgical versus post-catheter ablation complications (e.g., strokes, phrenic nerve injury, pulmonary vein stenosis).

### 3.3 Totally Thoracoscopic Hybrid Procedure Data Review

#### 3.3.1 Single Hospitalization Hybrid TT Maze Results

Reported single hospitalization 1-year HRS rhythm success rates have ranged from 60% to 82% [14, 25, 27, 28, 29, 30, 31]. De Asmundis *et al*. [32] have also reported their extended 1, 2 and 3 year results of 82%, 79% and 79% respectively. While most series report that failures of treatment are mostly non-atrial fibrillation (i.e., atrial flutter and atrial tachyarryhtmias) [27, 30, 33], independent predictors of recurrence have included female gender, in-hospital atrial fibrillation [14, 32], prolonged pre-op AF, and pre-op mitral regurgitation [14]. Attempts to restore normal sinus rhythm with repeat catheter ablations ranges from 1% [34] to 20% [27]. Although depressed EF has not been described as a predictor of recurrence and despite RCTs (CASTLE-AF [35], CAMERA-MRI [36]) that demonstrate the promise of catheter ablation in a depressed LVEF population, only a single published series with a TT Maze Hybrid approach in depressed EF patients is reported and it also reveals significant improvement in LVEF% with 61% HRS success at 32-month follow-up [37].

#### 3.3.2 Double (Staged) Hospitalization Hybrid TT Maze Results

Dunnington *et al*. [22] have reported the largest single center series of a 2-stage TT Hybrid approach. Ninety-seven percent of patients in their 410-patient cohort had non-paroxysmal AF and they report an 81% HRS success at 1-year, 76% at 2-years and 66% at 3-years follow-up. Additional centers have also reported encouraging 1-year HRS success results ranging from 93% [38], 89% [39], 88% [40], and 78% [41]. Eighteen-month 56% [42] and 65% 2-year HRS success [43] have also been reported. Finally, although no RCT is currently available, a meta-analysis of published studies has demonstrated a significant rhythm control advantage of the TT Hybrid approach when compared to endocardial catheter ablation alone (70.7% vs 49.9%, *p *< 0.001) [44] as well.

#### 3.3.3 Stand-Alone single Epicardial Surgical Ablation 

The CASA-AF multi-institutional randomized control study compared a single stage epicardial ablation approach with catheter ablation alone [45]. The study included 54 patients in the surgical arm and 60 patients in the catheter ablation arm. Overall, HRS success was 26% in the surgical arm and 28% in the catheter ablation arm with no statistically significant difference between the groups. The authors concluded that a single stage surgical procedure is not superior to catheter ablation in de novo long-standing persistent AF. Interestingly, these very low success rates in both arms of this prospective trial are significantly lower than many reported series. It is possible that the lower success rates are a function of monitoring with an implantable loop recorder (versus repeat Holter ECG) which would be more sensitive in detecting episodes of AF. Muneretto *et al*. [40] provide us with additional data in their prospective multicenter single-arm study which evaluated the outcomes of a single stage epicardial ablation or hybrid approach (if needed). They report 1-year HRS success of 75% and 88% in the isolated surgical and hybrid approaches respectively [40]. These data are more in concert with other reports as well, 78% HRS success with a median follow-up time of 866 days [41].

#### 3.3.4 Safety of the HTT Maze Approach

The reported rhythm control advantage of the TT Hybrid maze approach is often, however not always, associated with an increased morbidity and mortality when compared to isolated catheter ablation. Maesen *et al*. [33] report excellent freedom from complications, with no major complications (mortality, conversion to cardiopulmonary bypass, or phrenic nerve injury) in 64 consecutive patients who completed their hybrid approach. Others have also shown an excellent safety profile with no reported mortality [46] and reported freedom from stroke at nearly 3-years at 98.7% [14]. However, stroke rates as high as 3% [42] and mortality rates as high as 1.3% [22] have also been reported; 1–2% phrenic nerve injury is also reported [22, 42] and conversion to thoracotomy or sternotomy is reported from 4% [39] to 0.4% [22]. In summary, overall freedom from complications is reported to range from 100% [29], 92.4% [14], 93.9% [22], 86% [30], to 80% [32]. Stroke incidence is low but also reported at 0.5 per 100 patient years [14].

### 3.4 Hybrid Totally Thoracoscopic Summary

**Fig. 9.**
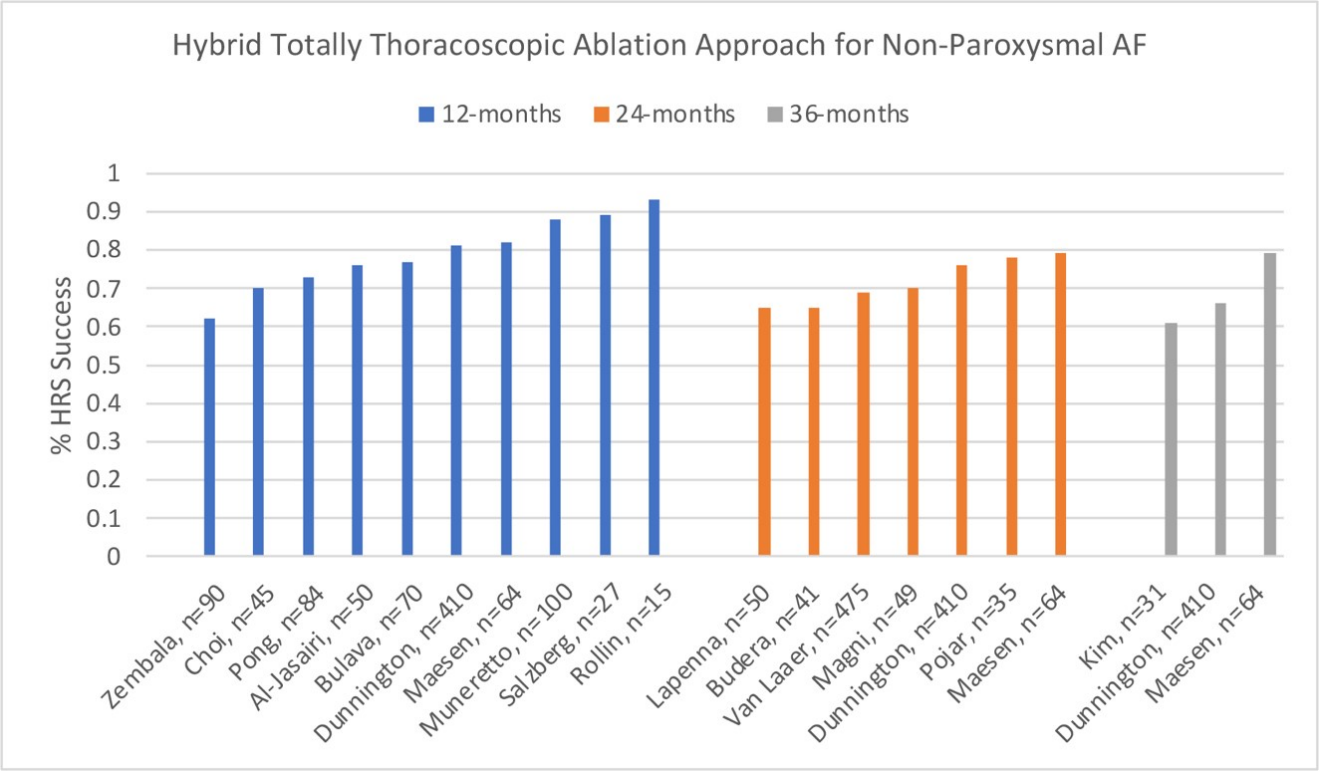
**Graphical Summary of Totally Thoracoscopic Hybrid HRS Success rates**.

In summary, the HTT Maze ablation approach has the potential to increase the patient response via an increased ablation lesion set that more closely mimics the Cox-Maze III/IV and is reported to have a wide range of success for restoration of normal sinus rhythm and freedom from atrial fibrillation (Fig. 9), and while select centers have reported excellent freedom from complications, in general the TT Hybrid approach confers approximately a 5% complication risk.

## 4. Programmatic Considerations

Treatment of AF through hybrid approach requires interdisciplinary collaboration. There are several questions which each team will need to consider as they develop a new program. Initially, the procedure was proposed as a concomitant epicardial and endocardial approach. Company statistics indicate that most procedures are now performed as a two-staged approach, with the epicardial portion occurring between 30–45 days prior to the endocardial ablation [*unpublished AtriCure™ data*]. There are several theoretical advances to this approach including coordination of OR time for busy subspecialties, allowing resolution of inflammation prior to mapping for potentially more accurate results, and separation of potential complications including bleeding.

The ideal initial approach to management of non-paroxysmal atrial fibrillation is also yet to be clearly defined. While newer programs may primarily treat non-responders to anti-arrhythmic or catheter ablations with HC or HTT approaches, many programs have evolved over time to refer patients earlier in their disease process, including “de novo” patients who prefer a comprehensive approach. Both referral patterns are appropriate and guided by recommendations from the Heath Rhythm Society (HRS) and the Society of Thoracic Surgeons (STS) for hybrid or stand-alone surgical ablations [17].

At initial consultation, a thorough review of common causes of atrial fibrillation should be investigated and modifiable risk factors should be addressed. A history and physical, including a focus on potential red flags or contraindications for the procedure is obtained. For example, a history of severe pericarditis or uremia may be indicative of adhesions in the pericardial space which may preclude pericardioscopic or thoracoscopic/VATS approaches. Prior heart surgery is considered by many to be a contraindication to the either off-pump epicardial ablation approach. The date and extent of prior catheter ablations should be noted. Some surgeons prefer to wait 3–6 months following the most recent CA to attempt navigation of the pericardial space due to inflammation. A transthoracic or transesophageal echocardiogram performed within the prior six months should be reviewed to rule out concomitant valvular pathology and evaluate chamber size. A CT of the chest is helpful if consideration will be given to concomitant appendage ligation through thoracoscopic approach. Teams will need to determine appropriate body mass index for hybrid ablation referrals, and for consideration of appendage ligation. Initially, it may be favorable to avoid morbidly obese patients for technical reasons. Within our own practice, we do not have an absolute contraindication point, but rather consider each case and approach individually.

Management of the left atrial appendage (LAA) is a key component of any successful multidisciplinary atrial fibrillation program. Once the surgical ablation has been completed, an epicardial clip (AtriClip AtriCure, Inc., Mason, Ohio) may be applied through several approaches including concomitant left thoracoscopic or mini-thoracotomy. As compared to other appendage devices, such as the Watchman or Lariat, the success of complete closure is similar (>90%) [14, 47]. No head-to-head trial exists between approaches to management of the left atrial appendage, but there is data that the LAA is an important source of arrhythmogenicity as well as thrombogenicity [10] and thus a theoretical advance of surgical clip in some settings. Additionally, although not specifically comparing this population, the LAOS III trial showed a potential benefit in patients with AF undergoing cardiac surgery, with LAA closure resulting in a lower risk of stroke or systemic embolism, with the continuation of oral anticoagulation [48]. Presently, approximately 80% of HC procedures include left atrial appendage management concomitantly with the surgical ablation [*unpublished AtriCure™ data*].

One point that is not well-define is the amount of time which anticoagulation should be held prior to and after the surgical ablation. In our practice, one dose of anticoagulation is held preoperatively and if there are no signs of bleeding the patient’s home dose of anticoagulation is resumed the evening of surgery. Decision for discharge or removal of surgical drains is not standardized and there are variations in practice regionally.

## 5. Discussion

Stand-alone and hybrid surgical ablation for PAF is currently endorsed as a IIB indication from HRS and a IIA indication for persistent and long-standing persistent AF [17].

Like many discussions regarding surgical ablation, the lack of definition as to what exactly is a hybrid procedure is lacking and imprecise. All surgical ablation is not a Maze procedure, yet physicians will use this term to apply to many lesion sets which have varying *expected* outcomes and rates of complications. For instance, similar outcomes for a transdiaphragmatic approach be expected for subxiphoid approach to HC ablation? Should providers use a different term to distinguish when the left atrial appendage is ligated thoracoscopically concomitantly with HC ablation? Is there added value to ablate the Ligament of Marshall when it’s under direct visualization and (hypothetically) adds little risk to the procedure at that stage? Is it “safe” to stop oral anticoagulation following confirmation of left atrial appendage closure with epicardial devices? When should patients be discharged following HC or HTT ablation? All these questions are still unknown and require further well-designed research to definitively answer. Guidelines to direct programmatic practices are lacking for HC and HTT ablations.

Lastly, the definitions of “success” in surgical, catheter, or hybrid ablation needs reconsideration. Most (if not all patients) would likely consider a significant reduction in the burden of AF a successful intervention, yet definitions of procedural success or failure hinge on seconds of recurrence of atrial fibrillation.

## 6. Conclusions

In summary, the posterior wall of the left atrium is increasing recognized as a substrate for non-paroxysmal atrial fibrillation. Both stand-alone and hybrid surgical approaches may be utilized as part of a comprehensive heart team approach to improve outcomes and reduce the burden of AF in this population. Further data are needed to identify predictors of long-term success with each approach.
